# Assessment of parasite virulence in a natural population of a planktonic crustacean

**DOI:** 10.1186/s12898-019-0230-3

**Published:** 2019-03-14

**Authors:** Eevi Savola, Dieter Ebert

**Affiliations:** 1 Department of Environmental Sciences, Zoology, Basel University, Vesalgasse 1, 4051 Basel, Switzerland; 2 Present Address: Institute of Evolutionary Biology, School of Biological Sciences, The University of Edinburgh, Ashworth Laboratories, Edinburgh, EH9 3FL UK

**Keywords:** Host-parasite interactions, Virulence, Castration, *Daphnia*, *Pasteuria ramosa*

## Abstract

**Background:**

Understanding the impact of disease in natural populations requires an understanding of infection risk and the damage that parasites cause to their hosts (= virulence). However, because these disease traits are often studied and quantified under controlled laboratory conditions and with reference to healthy control hosts, we have little knowledge about how they play out in natural conditions. In the *Daphnia*–*Pasteuria* host–parasite system, field assessments often show very low estimates of virulence, while controlled laboratory experiments indicate extremely high virulence.

**Results:**

To examine this discrepancy, we sampled *Daphnia magna* hosts from the field during a parasite epidemic and recorded disease traits over a subsequent 3-week period in the laboratory. As predicted for chronic disease where infections in older (larger) hosts are also, on average, older, we found that larger *D. magna* females were infected more often, had fewer offspring prior to the onset of castration and showed signs of infection sooner than smaller hosts. Also consistent with laboratory experiments, infected animals were found in both sexes and in all sizes of hosts. Infected females were castrated at capture or became castrated soon after. As most females in the field carried no eggs in their brood pouch at the time of sampling, virulence estimates of infected females relative to uninfected females were low. However, with improved feeding conditions in the laboratory, only uninfected females resumed reproduction, resulting in very high relative virulence estimates.

**Conclusions:**

Overall, our study shows that the disease manifestation of *P. ramosa,* as expressed under natural conditions, is consistent with what we know from laboratory experiments. However, parasite induced fecundity reduction of infected, relative to uninfected hosts depended strongly on the environmental conditions. We argue that this effect is particularly strong for castrating parasites, because infected hosts have low fecundity under all conditions.

## Background

Along with other environmental factors, parasitic infections—and especially virulence, the fitness costs from such infections—play a key role in limiting and controlling host populations [1–5]. The study of infectious disease virulence in humans [1, 6] and diverse animal populations [7–10], has revealed a vast range of consequences for individual hosts and host populations. For example, the degree to which a parasite regulates host population density depends in a non-linear way on how it influences individual host fecundity and survival: parasites that mainly reduce host fecundity suppress host density to a greater degree than parasites that induce host mortality [11, 12]. Therefore, understanding the expression of virulence in individual hosts is vital for our understanding of the processes that govern host population and disease dynamics in the natural world [13]. However, assessing virulence under controlled laboratory conditions may not accurately reflect how the host suffers from infections under natural conditions, which are not controlled and are often highly variable. By better understanding how disease symptoms are expressed under natural conditions, we can gain a better quantitative understanding of the causes and consequences of wildlife epidemics, which could, for example help design biological pest control programs [14, 15].

For several reasons the effect of parasites on individual hosts is difficult to assess under natural conditions. First, parasite-induced host mortality and host fecundity require prolonged observation of individual hosts, and infected and uninfected hosts need to be compared to observe the effect of infection. This comparative or “relative” aspect of defining virulence is important, although frequently overlooked [16, 17]. Without it, we cannot distinguish parasitic effects on host wellbeing from other effects, such as environmental conditions, which may affect the wellbeing of infected and uninfected hosts alike. For example, resource limitation may increase mortality and decline fecundity in infected and uninfected hosts equally. Second, because infections may not be easily detected (for example, in recently infected animals), the infection status of individual hosts may not be accurately assessed. Third, infections may increase mortality, thus removing from the population those animals that show the strongest symptoms and consequently reducing estimates of virulence in the surviving population. Fourth, as infections by multiple parasites are common under natural conditions, but not always easily detected, the link between disease symptoms and a specific parasite is not always clear [10, 18]. Finally, infected individuals may not be “typical” for the study population. Hosts in poor shape, for example, may become infected more easily and appear therefore to be more strongly affected by the disease than other hosts. Taken together, these issues make working with natural populations challenging. Here, we undertake a study comparing estimates of disease parameters caused by a bacterial parasite in a natural zooplankton population with results from laboratory experiments under controlled conditions.

*Pasteuria ramosa* is a common, obligate, bacterial endoparasite of the planktonic crustacean *Daphnia* (reviewed in [19]). In the laboratory, its waterborne spores can infect animals of any age, class, and sex that are exposed to them, although susceptibility does vary [19, 20]. Infection changes the host’s body colour, and causes loss of transparency, castration of females, enhanced body growth (gigantism) and increased mortality [19]. Under controlled laboratory conditions, infected juveniles lose about 90 to 100% of their residual life-time reproductive value (relative to uninfected females), whereas adults lose about 80 to 90% [9, 21–26]. These observations, however, contrast markedly with field studies on wild-caught animals. One study looking at host reduction of fecundity in three host species from three ponds in England estimated a decrease of about 8% in the likelihood of carrying a clutch in *Daphnia pulex*, while even weaker and non-significant effects were reported for *D. magna* and *D. longispina* [27]. A study of a Belgian *D. magna* population, found a significant, but weak, fecundity reduction in association with *P. ramosa* infections [28]. In a *D. magna* population in northern England, Duncan and Little [29] observed similar weak effects, but did not quantify them. Likewise, a study of *D. longispina* reported a weak and non-significant reduction of fecundity upon infection with *P. ramosa* [30]. How can this discrepancy—between fecundity reductions of over 80% in laboratory experiments and small fecundity reductions in the field—be explained? Additionally, how do other disease symptoms compare between the field and laboratory? Here we attempt to answer these questions. Based on our observations of experimental infections and disease progression in controlled laboratory conditions, we suggest and test the following predictions:

- First, in advanced infection stages, females are castrated by the parasite, i.e. they carry no sexual or asexual eggs [19]. We expect this to hold true even under improved feeding conditions, where most infected females usually do not reverse castration, although some may produce a few small clutches after being initially castrated [26, 31].
- Second, in the laboratory, *P. ramosa* symptoms (colour change, castration, gigantism, loss of transparency) appear about 10 to 20 days after infection [19]. Time to maturity takes about 6 to 12 days (longer under poor feeding conditions). Thus, we expect that infected juveniles will not show disease symptoms on the day of their capture. In addition, since their infections will be recent, we expect their disease symptoms to show up towards the latter end of the time spectrum (i.e. closer to 20 days than 10). Juveniles caught in the field and kept under good feeding conditions are, thus, more likely to produce offspring before castration than older, larger animals, whose infections are probably older as well.
- Third, in the laboratory *P. ramosa* causes chronic disease. Thus, we expect infection prevalence in natural populations to increase with host age, which in *Daphnia* can be recognized by their larger size, as they grow indeterminately. This prediction assumes that the parasite neither causes increased mortality in older animals nor that it influences host body size. Both these assumptions are violated by this system. However, since parasite-induced mortality and parasite-induced gigantism start only later in an infection [19, 32], we predict that the positive body size—prevalence relationship will be clearly visible at least for smaller (younger) size classes.
- Fourth, in the laboratory, both sexes and all age classes are susceptible to *P. ramosa* infection, although younger age classes are overall more susceptible to parasite exposure [20, 33]. We predict that both sexes and all age classes (using size as a proxy) are susceptible to *P. ramosa* in the field.

To test these predictions, we collected *D. magna* from a natural population during a *P. ramosa* epidemic and kept them under good feeding conditions in the laboratory. They were housed individually in artificial medium to prevent any new laboratory infections. Our findings largely coincide with laboratory experiments and explain why previous field studies underestimated the parasite’s effect on virulence.

## Materials and methods

Our study site was Ägelsee, Switzerland, a freshwater lake without fish (47°33′28.6″N, 8°51′43.3″E, approximately 1.7 ha, maximum depth 3.5 m) [34]. In this eutrophic lake, three *Daphnia* species coexist: *D. magna, D. pulex* and *D. curvirostris*. Monitoring of these *Daphnia* populations for *P. ramosa* infections since autumn 2010 has revealed a major epidemic of *P. ramosa* every summer in the *D. magna* population, whereas the other two *Daphnia* species have never shown any sign of this infection [34] (D. Ebert, ongoing longitudinal sampling). Other *D. magna* parasites have not been observed above a 0.5% prevalence in any sample. For the current study, we sampled *D. magna* during the entire summer season (April to October) to evaluate the prevalence of *P. ramosa*. On two dates, June 7, (first sampling) and June 28, (second sampling) 2015, we collected large samples of *D. magna* to assess infection risk and parasite virulence. Earlier samples (from May 2015) had shown too low of a parasite prevalence, while from July onwards, prevalence was too high to allow for a meaningful comparison of infected vs. uninfected hosts. Sampling was done from a platform close to the deepest part of the lake; using a 200-µm zooplankton net, we collected 500 to 1000 *D. magna* individuals from all depth layers by taking several vertical hauls from the water column (bottom to top at the deepest place). *D. magna* were then transferred into a 1:1 mix of filtered (20 µm mesh size) pond water and artificial *Daphnia* medium (ADaM; Klüttgen et al. [35]; modified by using only 5% SeO_2_). Using ice, we reduced the temperature from about 25 to about 15 °C. Samples were transported to the laboratory and processed the same day within 8 h after catching.

In the laboratory, individual *D. magna* were measured with a stereomicroscope for body length from the tip of the head to the base of the tail spine. The first sampling showed a fairly uniform distribution of body lengths, whereas in the second sampling, small individuals were strongly underrepresented. Therefore, we enriched the second sample with small juvenile animals taken from the same field sample as the other animals. Adult *Daphnia* reproduce (at 20 °C) about every 3–4 days. Eggs (asexual clutches and resting eggs) then remain with the mother for 3–4 days, allowing us to assess the female’s reproductive condition. For each animal, we recorded sex (male/female), infection status (1/0, as judged by visible symptoms), presence of ephippia (1/0) or presence (1/0) and number of eggs in the brood pouch. The maturity (juvenile/adult) of females was classified by the relative length of the longest (least caudal) abdominal process, which encloses the female’s brood pouch, and in males by the appearance of a wider gap in the upper ventral rim of the carapace.

Individual animals were each placed into a 100-mL clear glass jar filled with about 80 mL ADaM and placed in an incubator (20 °C ± 0.5, 16:8 h L:D). For the first sampling, 309 individual females and 25 males were collected. For the second sampling, 408 females were collected; males were excluded in this sampling analysis due to very low numbers in the population. Jars and medium were changed at least every 7 days. Daily feeding, using chemostat cultured green algae *Scenedesmus* sp., was adjusted for life-stages to allow for ad libitum feeding, with small amounts of food still left over by the next day’s feeding. This required about 2 million cells per jar for very young animals to 15 million cells for very large animals. All animals were checked daily for survival, signs of infection, and number of offspring released. Offspring were counted, and the mothers transferred to new jars. Infections were recognizable by a clearly visible change in body colour (the normally semi-transparent body turns opaque brownish-yellowish) and the absence of eggs in the brood chamber or resting eggs (ephippia). The parasite is also visible in the body cavity of the transparent host under a stereo-microscope at 100× magnification. No evidence of other parasites was observed during the study, although some epibionts (*Vorticella* sp.), which did not interfere with host well-being, were observed attached to the host carapace.

We examined those *D. magna* individuals that died during the 3 weeks post-collection with phase contrast microscopy at 200 to 400× magnification to look for the presence of the typical blood stages of *P. ramosa*. For animals that died before clear signs of infection were visible, we estimated the time to visible infection by considering the developmental stage of their parasites. *D. magna* individuals hosting parasites in the grape-seed stage were calculated to take five more days to show external signs of infection, while for hosts with cauliflower stage parasites we added 10 days [36].

All statistical analysis was conducted using R software version 3.1.1 (R Core Team, 2014) and the R-package MASS [37]. We used Fisher’s exact test to determine the relationship between maturity (juvenile/adult) and infection levels (1/0), as well as the relationship between sex (male/female) and infection levels. An independent 2-group Mann-Whitney U Test was conducted on the numbers of offspring per clutch for uninfected and infected females and on the number of eggs at sampling time for uninfected and infected females. Generalized Linear Models (GLM) were used with explanatory variables of body length (mm), sampling date (factorial with two levels) and the interaction between the two. We tested assumptions about each specific model (e.g. dispersion or distribution, depending on the type of the statistical model in question). Logistic regression with quasibinomial distribution was used to account for the variation in infection status (1/0) and for the variation in clutch presence (1/0). Total fecundity levels were analysed with a negative binomial GLM. Time to infection was analysed using linear regression.

## Results

On capture day of the first sample, 15 out of 309 females (4.8%) showed clear signs of infections. At the end of the laboratory observation period, total infection levels in the first sample were 30.8%. On capture day of the second sample, 156 of 408 females showed clear signs of infection (38.1%). This number rose to 88.0% at the end of the observation period.

### Host fecundity and virulence

**Fig. 1:**
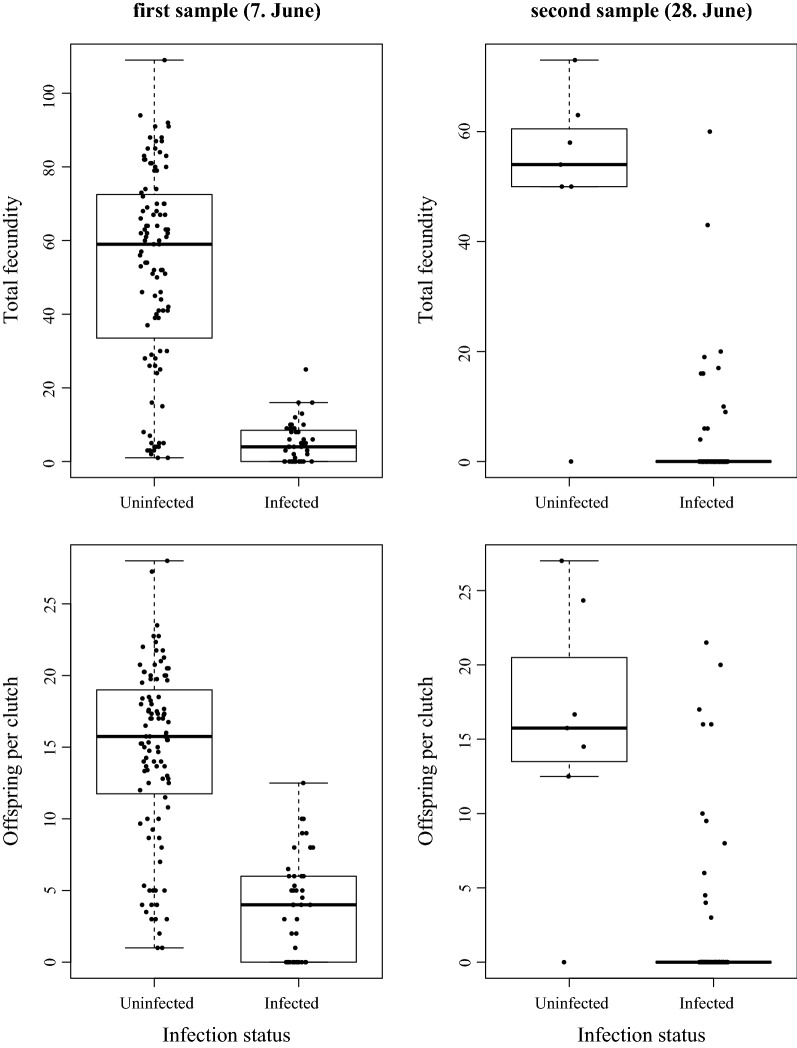
Offspring of uninfected and infected *D. magna* females from the first (left panels) and second (right panel) sampling produced after the laboratory observation period. Differences are highly significant (Total fecundity: first sample: Mann-Whitney U Test, W = 4042.5, p = 3.4 × 10^−16^; second sample: W = 1179, p = 4.6 × 10^−13^; Number of offspring per clutch: first sample: W = 3976, p = 3.6 × 10^−15^, second sample: W = 1066, p = 1.8 × 10^−9^)

On capture day of the first sample, none of the 15 females with signs of infection carried offspring in their brood pouches, whereas 17 of the 294 apparently uninfected females did. This difference in fecundity was not significant (Table 1, Wilcoxon rank sum test, W = 1064, p = 0.179). Additionally, none of the infected females carried resting eggs (ephippia) in the first sample, compared to 10 uninfected females that did. On capture day of the second sample, only one female captured carried eggs in her brood chamber; she was uninfected (Table 1). Thus, as earlier field studies have shown, the overall quantitative effect of the parasite on host fecundity was small. However, this changed after these females were brought into good feeding conditions in the laboratory. Uninfected *D. magna* produced more offspring per clutch and more offspring in total during the observation period than the infected female *D. magna* in both samples (Fig. 1).

**Table 1:** Summary of parameters for *D. magna* females: first and second samples

	First sample (7. June 2015)	Second sample (28. June 2015)
Total number of collected females	309	408
Females on sampling day (= day of capture)		
Percentage infected females on day of capture	4.8%	38.2%
Number of females with eggs		
Uninfected	17	1
Infected	0	0
Average number of eggs in broodpouch		
Uninfected	0.383 (± 1.136)	0.027 (± 0.001)
Infected	0 (± 0)	0 (± 0)
Number of females with ephippia		
Uninfected	10	0
Infected	0	0
Average body length		
Uninfected	2.37 mm (± 0.65)	2.10 mm (± 0.41)
Infected	3.26 mm (± 0.40)	3.21 mm (± 0.40)
Females at the end of the observation period		
Percentage infected at the end of observation period	40.1%	88.0%
Number of days for infection to be recognized	13.7 (± 4.9)	10.1 (± 6.79)
Number of clutches prior to parasitic castration	0.89 (± 0.8)	0.09 (± 0.4)

Where appropriate, means ± standard deviations are given

### Host sex and stage

**Fig. 2:**
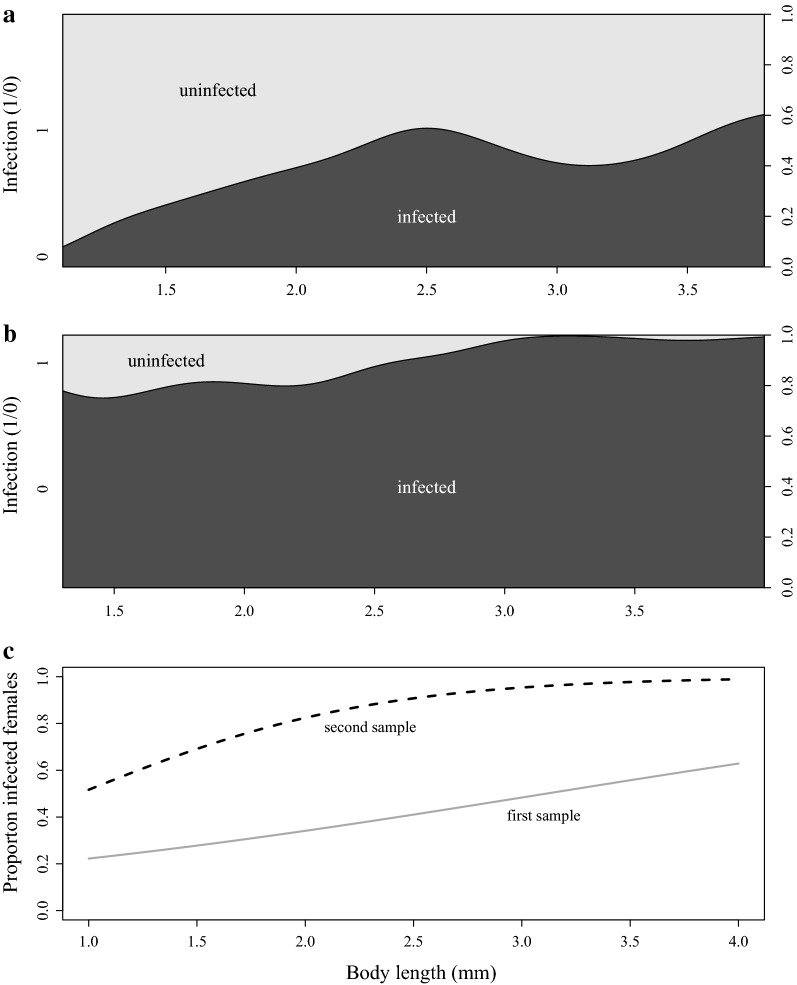
Density plot for infection (dark shading) by the end of the observation period, by body length at capture for the first (**a**) and the second sample (**b**), and the predicted relationship between body length at capture and infection levels fitted with a quasibinomial model (**c**). Solid and dashed lines represent the first and the second sample, respectively. Body size at maturity was on average 2.6 mm in both samples

Adults were more commonly infected than juveniles by the end of the observation period, but this difference was only significant in the second sample (prevalence first sample: juveniles: 35.2%, n = 159, adults: 45.3%, n = 150, Fisher’s exact test, p=0.082; second sample: juveniles: 80.6%, n = 222, adults: 96.8%, n = 186, Fischer’s exact test, p = 2.01 × 10^−7^). Overall, infection prevalence increased with host body length, but individuals of any body length were more likely to be infected in the second sample (Fig. 2, Table 2A). Moreover, the increase in infection prevalence with host body length was close to be monotonic for the second sample, but not so for the first sample (Fig. 2a, b). Thus, it is not clear if a simple chronic disease model is appropriate to describe this distribution. The deviation from a monotonic increase may have stemmed from parasite-induced host gigantism and from an increased mortality of older (larger) infected hosts. Clearance of *Pasteuria* infection has not been reported so far. Figure 2 indicates that even the smallest animals could be infected. It is important however, to note that our sample did not contain newborn hosts (size class < 1.0 mm), as they were not found in our samples. *Pasteuria* is only known to transmit horizontally; thus, newborn are uninfected at birth [19].

**Table 2:** Statistics for the effects of sampling date, body length (mm) and their interaction on disease parameters

A. Likelihood of infection (Fig. 2c)	Estimate	Standard error	T value	Pr (> |t|)
Constant	− 1.843	0.449	− 4.106	*4.5* *×* *10*^*−5*^
Sampling date (28 June 2015)	0.432	0.799	0.541	0.589
Body length	0.592	0.176	3.364	*0.00081*
Sampling date : body length	0.886	0.351	2.521	*0.012*

B. Time taken to show infection (Fig. 3)	Estimate	Standard error	T value	Pr (> |t|)
Constant	21.491	1.816	11.834	*<* *2* *×* *10*^*−16*^
Sampling date (28 June 2015)	7.830	2.046	3.827	*0.00015*
Body length	− 4.098	0.690	− 5.937	*5.6* *×* *10*^*−9*^
Sampling date : body length	− 4.318	0.775	− 5.572	*4.2* *×* *10*^*−8*^

C. Total number of offspring produced prior to castration (Fig. 4a)	Estimate	Standard error	Z value	Pr (> |z|)
Constant	2.013	1.908	1.055	0.291
Sampling date (28 June 2015)	3.431	2.284	1.502	0.133
Body length	− 0.158	0.735	− 0.215	0.829
Sampling date : body length	− 2.428	0.932	− 2.605	*0.0092*

D. Probability of producing a clutch prior to castration (Fig. 4b)	Estimate	Standard error	T value	Pr (> |t|)
Constant	5.618	1.273	4.412	*1.4* *×* *10*^*−5*^
Sampling date (28 June 2015)	− 0.358	2.177	− 0.164	0.869
Body length	− 1.434	0.463	− 3.098	*0.0021*
Sampling date : body length	− 2.412	1.095	− 2.202	*0.0283*

A. Quasibinomial regression model fitted to analyse the likelihood of infection (0/1) (null deviance = 904.88, df = 715, residual deviance = 674.57, df = 712). B. General linear model (GLM) for the time (in days) taken to show infection (R-squared = 0.58, F_3,478_ = 220.1, p-value ≤ 2×10^−16^). C. Negative binomial generalised model for total number of offspring produced prior to parasitic castration (null deviance = 126.012, df = 226, residual deviance = 96.052, df = 223). D. Logistic regression model with quasibinomial distribution for the probability of producing at least one clutch prior to parasitic castration (null deviance = 456.58, df = 332, residual deviance = 170.43, df = 329). P-values in italic are statistically significant at the level α = 0.05

Both males and females became infected during the observation period. In the first sample, males had a somewhat higher (but not significant) prevalence at the end of the observation period (prevalence male: 56.0%, n = 25, females: 40.%, n = 309, Fisher’s exact test, p = 0.141). In the second sample, males were too rare to analyse.

### Body size and fecundity of infected females

**Fig. 3:**
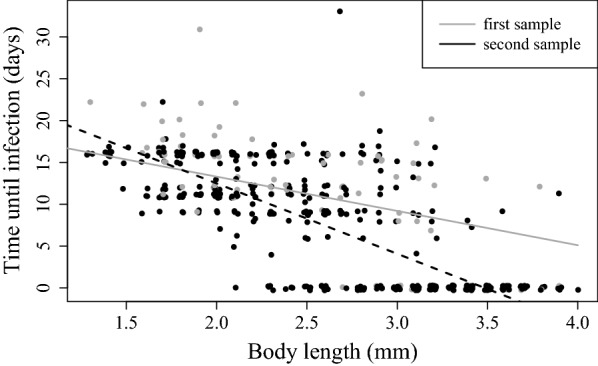
Relationship between body length at capture and time to visible signs of infection. Lines fitted with a GLM. Grey line and grey data points (grey circle) represent the first sampling and black line and black data points (black circle) represent the second sampling

As predicted, females scored as uninfected on capture day in both samples were, on average, smaller than infected females (Table 1, first sample: Wilcoxon rank test, W = 571.5, p = 1.264 × 10^−6^; second sample: W = 1578, p = 2.2 × 10^−16^). As we know from laboratory observations, *P. ramosa*-induced symptoms appear about 10 to 20 days after infection. Thus, disease symptoms are expected to show up a few days later in younger animals, whose infections are likely newer. Indeed, smaller and, thus, younger infected females showed, on average, a longer delay until signs of infection became visible than did larger females (Fig. 3, Table 2B).

**Fig. 4:**
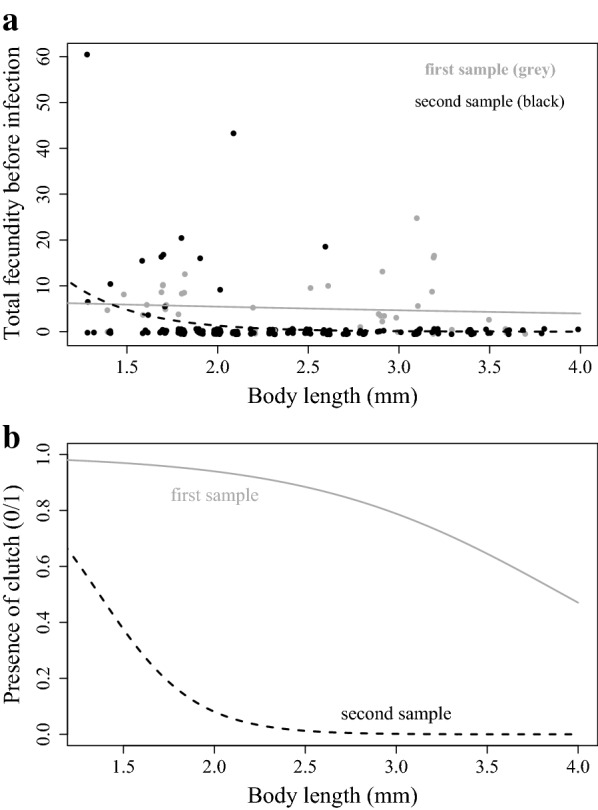
**a** Relationship between body length at capture and total fecundity of infected females prior to castration. Lines fitted with a negative binomial generalized model (Table 2C). B. Relationship between body length at capture and probability of infected females. **b** Producing at least one clutch prior to parasitic castration fitted with a GLM with quasibinomial distribution (Table 2D)

As host castration, as observed in the laboratory, generally happens only after about 15 to 20 days of *P. ramosa* infection, we predicted that smaller and, thus, younger *D. magna* from the field would be more likely to produce offspring under ad libitum feeding conditions than larger (older) individuals that probably carry more advanced infections. In the first sample, body length had no significant effect on total fecundity before castration, while in the second sample, body length correlated negatively with the infected female’s total fecundity (Fig. 4a, Table 2C). The likelihood of carrying a clutch before castration took place declined with increasing body length in both samples, and females from the first sample were more likely to have at least one clutch than females from the second sample (Fig. 4b, Table 2D).

## Discussion

### Parasitic castration, selection and the role of the environment

This study was motivated by the question of whether parasitic diseases manifest differently under natural conditions versus controlled laboratory conditions. Such a discrepancy would distort our interpretations of evolutionary ecology and the epidemiology of host-parasite interactions, as these are often based on results obtained under controlled conditions. To address this question, we collected hosts during an epidemic in a natural population, recorded life history and disease traits, and kept them in the laboratory under good feeding conditions. Within the limits of inference that this approach allowed, we conclude that the manifestation of the disease is consistent between field and laboratory conditions and that the apparent discrepancy in the effect of the parasite on relative host fecundity (a measure of virulence) is caused by the high sensitivity of relative fecundity on the environmental conditions (which differ between field and laboratory). Uninfected, but not infected females respond strongly to improved feeding conditions.

Parasite virulence is typically assessed as host fitness (or components of it) in infected hosts relative to uninfected hosts [17]. This relative nature of virulence thus depends on the condition of infected and uninfected animals alike, both of which are affected by environmental conditions. During the here studied epidemic, all hosts—infected and uninfected alike—suffered from limited resources. The overall fecundity of the *D. magna* population was very low at the time of both samples (0.38 and 0.027 asexual eggs per uninfected female for the first and second sample, Table 1). Indeed, the numbers for uninfected females showed no significant difference from those for infected females that carried either sexual or asexual eggs at the time of sampling. This result is consistent with earlier field studies of this system [27–29] that showed low and mostly non-significant estimates of relative virulence. Relative virulence estimates increased dramatically when the females were provided with sufficient food under laboratory conditions. While infected females still had very low fecundity, the uninfected females produced large clutches (Fig. 1). These results reflect published estimates from life table experiments for this system [9, 21–26]. Furthermore, they coincide with an experiment that reported that host starvation did not affect the ability of the parasite to castrate the host, but strongly affected host reproduction of those females that were able to reproduce [38].

If *Pasteuria* virulence under natural conditions is indeed much lower than laboratory experiments estimate, the potential of *Pasteuria* to select for resistance in the host population may be lower as well. To answer this question, we need to carefully evaluate the strength of selection and the response to it in natural populations. Conditions in the field change across the year and *Daphnia magna* populations are highly dynamic [39]. Parasites may even amplify such dynamics [40], as they reduce density and, thus, free up resources [29]. In temperate European regions, *Pasteuria* epidemics occur mainly during the warmest season of the year [27–29, 41, 42], which is consistent with the parasite’s good performance under high temperatures [23]. During this time, *D. magna* populations typically exceed their first density peak and have low clutch sizes [39]. Therefore, under natural conditions, *P. ramosa* epidemics often occur when host reproduction is low and we can expect that estimates of virulence based on relative fecundity will be generally much lower under these conditions than estimates for laboratory reared animals. On the other hand, resource levels in the field fluctuate over the summer and host population density will be affected by this. Furthermore, host density decreases as a consequence of the *Pasteuria* epidemics [28, 29, 41], freeing available resources for the surviving animals. Thus, throughout the summer, periods with improved resource levels occur and open opportunities for population recruitment. Our observations suggest that only uninfected females participate in this process, resulting in strong selection for resistant hosts that produce offspring. Thus, while fecundity reduction estimates of 80 to 95% in laboratory experiments [19] seem too high to reflect fecundity reduction in natural conditions, we believe that the parasite is likely to exert strong selection on their hosts nevertheless.

Laboratory studies have also shown that poor nutrition influences other disease traits in *D. magna*. When food is plentiful, infected female *D. magna* produce more parasite spores, show stronger gigantism, die earlier, and produce more offspring than poorly fed animals [21, 38]. However, our study design did not allow us to study any of these effects. Similar effects were also seen in other *Daphnia* infections and can profoundly influence parasite epidemiology [43].

### Manifestation of other traits of *Pasteuria* infections

Although we have focused above on castration as the key symptom of *Pasteuria* infections, these infections in *D. magna* are well characterized, and their progression from exposure to host death has been described in great detail [19]. We observed, for example, the typical time delay between the host’s exposure to the parasite and the onset of disease symptoms. Laboratory studies have shown that the time from exposure to expression of disease symptoms is about the same as the host’s maturation time. Large (old) hosts may have been exposed recently or longer ago, while small (young) infected females would have been infected more recently. We thus predicted that, at the time of sampling (capture), typical disease symptoms would only be visible in adult females as juvenile infections would be recent. We also predicted that the time to infection (time to onset of symptoms) would decline with body size. Both predictions were confirmed here (Fig. 3). We also expected to see this time span reflected in the animal’s ability to reproduce before castration occurred. Indeed, we found that smaller (younger) infected females were more fecund than larger (older) females before castration started (Fig. 4).

Consistent with experimental studies [44], we also observed infections in both males and females captured in the field. Also consistent with previous reports, we found that females of all size classes (a.k.a. all ages) can become infected, but with our study design we were unable to observe variation in susceptibility among age classes [20, 33, 45]. Our data partially support the prediction that prevalence generally increases with body size, as is expected for chronic parasitic infections that accumulate in the population with host age. Similar findings have been made in other host–parasite systems [45–52]. However, parasite-induced gigantism and infection related mortality can distort the relationship between body size and prevalence.

Although the increase in disease prevalence with host size may occur simply because infections accumulate with increasing host age (older hosts have had more time to contract the disease than younger animals), other factors may influence this relationship. For example, larger *D. magna* require more food and are more likely to encounter parasite spores in the sediment and water for a number of reasons, including food filtering rates [53, 54], food density effects with body length [55], and body length in relation to gut size and clearance rate [56]. On the other hand, Izhar and Ben-Ami [20] have shown that susceptibility declines with age (and size) in the *D. magna*–*Pasteuria* system. More refined experiments are needed to test these possibilities, as our data do not allow us to address these questions.

## Conclusion

**Fig. 5:**
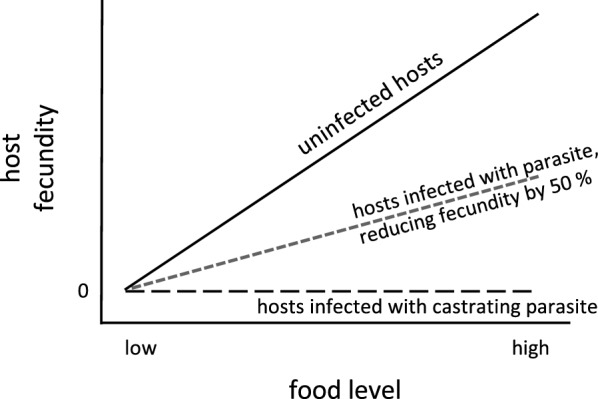
Schematic relationship between host fecundity and food level for infected and uninfected females. Fecundity increases with greater food levels. This is also seen for females infected with non-castrating parasites (here shown for a parasite reducing fecundity by 50%), but not for females infected with castrating parasites

To understand the evolutionary ecology and epidemiology of infectious diseases, we must know how disease symptoms express themselves under natural conditions. This expression is influenced by the length of the time from exposure/infection to disease outbreak (prepatent period), the effect of the infection on host morbidity and mortality, and on the transmission of the parasite. Our study explored whether the assessment of some of these parameters under laboratory conditions is consistent with the way the disease manifests itself under natural conditions. Although this type of study has rarely been undertaken for any system (but see [57]), it is essential for understanding disease spread. Our results, obtained from a natural population, support findings about disease progression obtained under controlled conditions. What did become clear, however, was that although parasitic castration is expressed under natural conditions, the quantification of this parasite driven fecundity reduction, relative to the fecundity of uninfected hosts, depends strongly on environmental conditions, which can vary between poor conditions in the field and the typically good experimental conditions. As environmental conditions decline, uninfected hosts reproduce less, while infected hosts mostly stop reproducing after castration (Fig. 5). Thus virulence, expressed as relative fecundity, declines with declining environmental quality. This phenomenon likely exists in other parasitic castrators systems as well. For non-castrating parasites that reduce only a fraction of the infected host’s fecundity compared to uninfected hosts, this phenomenon would be less pronounced. For example, if hosts infected with non-castrating parasites always, produce half as many offspring as uninfected hosts, relative virulence would be independent from the environment (Fig. 5). For example, in the *D. dentifera*–*Metschnikowia* system, the fecundity of both infected and uninfected animals increases with increasing food level [58], forming a clear contrast to the *Daphnia*–*Pasteuria* system studied here. Castrating parasites are rare in the *Daphnia* system [12], but are widespread across animal and plant taxa [59, 60]. Whether the different levels of environmental sensitivity between castrators and non-castrators influence the evolution and epidemiology of the system is a question that needs to be resolved in the future.
